# Filth Flies, Flowers and Food: Pollination by Flies (Calliphoridae) Does Not Affect the Strawberry Microbiome

**DOI:** 10.1007/s00248-026-02741-2

**Published:** 2026-03-25

**Authors:** Jonathan T. D. Finch, Markus Riegler, James M. Cook, Laura E. Brettell

**Affiliations:** 1 Hawkesbury Institute for the Environment, Western Sydney University, Penrith, NSW 2751 Australia; 2 Tasmanian Institute of Agriculture, University of Tasmania, Sandy Bay, TAS 7005 Australia; 3 School of Science, Engineering and Environment, University of Salford, Manchester, M5 4WT UK

**Keywords:** Blow fly, microbiome, environment, 16S, strawberry, fruit, flower

## Abstract

Blow flies can be used as alternative pollinators to honeybees for several crops. However, they can potentially vector harmful bacteria to surfaces on which they land. Therefore, there is a risk that blow flies may contaminate flowers, and thereby fruit, with human pathogens. Growers, supermarkets and consumers may therefore have concerns about food safety. To address these concerns, we asked: (1) do fly visits result in altered bacterial communities on flowers, and if so, (2) do these altered communities persist to the fruit stage? We exposed strawberry flowers to “clean” blow flies and flies that had contacted a common environmental contaminant (cow manure). Using a 16 S rRNA gene amplicon sequencing approach, we evaluated the impact of the blow fly visits on the flower and fruit microbiomes. We found that the fruit microbiome was not impacted by our treatments, specifically, no shared amplicon sequence variants were found in both the manure and the resulting fruit that developed after pollination by a manure-exposed fly. This suggests that fly pollination is unlikely to pose a risk to human health. Our study may help to alleviate concerns from growers and consumers of using flies as managed pollinators in the agrifood system.

**Supplementary Information:**

The online version contains supplementary material available at 10.1007/s00248-026-02741-2.

## Introduction

Many crops experience a pollination deficit, with implications for economic prosperity and human health [32, 47]. To reduce this pollination shortfall, various species of blow flies (Diptera: Calliphoridae) have been proposed and trialled as alternative pollinators to bees [7]. Blow flies show excellent potential as pollinators, due to the propensity of adult blow flies to visit flowers to obtain nectar and pollen. Blow flies can also be shipped and handled easily and reared very economically on cheap waste products, such as offal. However, blow flies are widely known to vector potentially harmful bacteria, including *Escherichia coli* and *Salmonella* species, to humans [4, 12, 13, 20, 25]. The risk of bacterial contamination may therefore deter growers from using blow flies as crop pollinators.

The microbes present on adult blow flies are strongly influenced by environmental factors. At eclosion, adult blow flies typically have very low bacterial diversity in their microbiome, which may be largely dominated by maternally inherited endosymbionts [20]. These obligate insect endosymbionts typically pose no known health risk to humans [21]. However, after eclosion, much of the external microbiome of blow flies is derived from their environment and resources that they choose to visit. These resources are likely to include all manner of decomposing materials, like sewage, faeces and carrion, which can potentially harbour pathogenic species [4, 13, 20, 35, 37].

Besides developing in and visiting rotting organic materials, blow flies commonly visit flowers to obtain nectar and pollen [10, 36, 42]. Blow flies can transmit a variety of microorganisms between surfaces, including pathogens that may be harmful to humans [4, 13, 20]. Therefore, it is possible that blow flies may transfer pathogenic bacteria onto flowers whilst feeding.

Many kinds of non-pathogenic bacteria are naturally present on flowers, where they consume soluble carbohydrates, amino acids and waxes [26, 30, 41]. Floral microbes are often derived from the soil and air [31, 45] or from other vegetative plant tissues, including pollen [22, 33, 48]. Microbes are often present on flowers even before flower anthesis, albeit at relatively low levels [2, 45]. After anthesis, microbial incidence and abundance can increase exponentially [2, 39, 48], and these changes are driven, at least partly, by pollinator visitation [3, 15, 22, 48]. Some microbes, such as yeasts, may be well adapted to colonising and reproducing on flowers, but non-flower adapted species may struggle to persist there [16]. Several factors, including floral morphology, high UV radiation, low moisture levels and competition from flower specialists may prevent the establishment of such non-adapted species [9, 43, 48, 49].

The fruit microbiome is distinct from that of the flower, but often shares some fungal and bacterial components [1, 38]. At least part of the fruit microbiome is inherited from the flowers. However, environmental filtering, as well as plant morphology, also plays an important role in shaping the unique fruit microbiome [38].

The microbes present on fruit can also be influenced by the agrifood system in which they are grown. For example, the fruit microbiome was found to be significantly different in organic vs. conventional apple orchards [1]. Fruits may be subject to contamination by bacteria, fungi and viruses through a variety of sources including animals, water, soil, machinery and human handling. During production, fruits, including strawberries, can become colonised by opportunistic pathogens and bacteria carrying antibiotic resistance genes [19, 27, 50]. The contamination of frozen strawberries by norovirus in Germany in 2012 impacted more than 10,000 people [29]. Freezing and storage of strawberries has a minimal effect on the survival of pathogens on berries [11, 24], highlighting the importance of preventing contamination at all stages during production [27].

There have been many studies of the bacterial communities spread by blow flies, as well as many attempts to assess the drivers of floral and fruit microbial communities. However, no studies have assessed the impact of blow fly pollination on the fruit microbiome and food safety. To address this knowledge gap, we attempted to answer the following questions, (1) do visits by blow flies result in altered bacterial communities on flowers, and, if so (2) do these altered communities persist to the fruit stage? In doing so, we aimed to give growers more confidence around the use of blow flies as alternate pollinators, and thereby, improve pollination security.

## Methods

### Experimental Setup

We choose to use strawberry as our model system for three reasons. Strawberries (*Fragaria × ananassa* Duchesne variety “Red Rhapsody”) are attractive to blow flies and they are commercially significant both locally and globally, with Australia producing in excess of 80,000 tonnes of strawberries per year (Hort Innovation [17]), . In addition, strawberries are one of the fastest growing fruits, developing from flower to berry in around 21 days under ideal conditions (JF, personal observation). This means that pathogenic bacteria deposited on the flower must only survive for a short period of time before potentially being consumed.

The experiment was conducted in November and December 2020 at Western Sydney University, Hawkesbury Campus, Richmond, NSW, Australia. Strawberry plants were purchased from Sweet’s Strawberry Runners (Queensland) and grown from runners in pots in a shade house on campus using a commercial potting mix. During the propagation and maintenance phase, watering was conducted using overhead irrigation for 5 min in the morning and afternoon. Prior to the flower opening, strawberry flowers were bagged using very fine nylon mesh bags (10 × 15 cm) to prevent visitation by all insects. The strawberry plants were then moved into a research glasshouse. The glasshouses were maintained at 21 °C and a relative humidity of 50–60%. Four whole strawberry plants were placed into 10 BugDorm™ 2F1M Cages (W60 x D60 x H60 cm) on two raised benches. Tap water was provided daily via a watering can into saucers in which the potted strawberry plants were sitting.

**Fig. 1:**
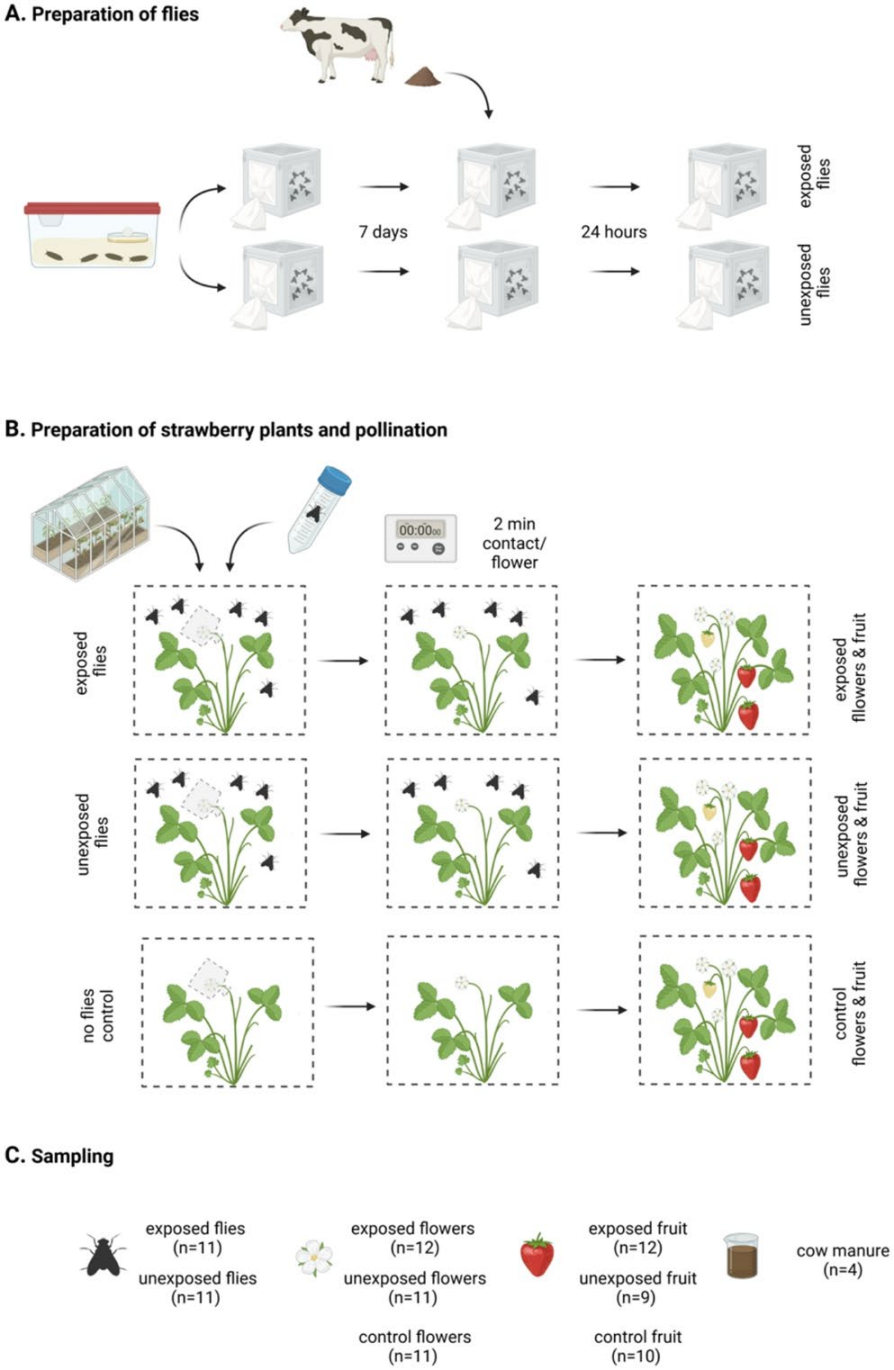
Experimental setup. Briefly, flies were prepared by rearing from pupae in plastic containers with wet sand placed into two cages. Adult flies were maintained with a sugar solution food source for seven days. One of the cages was then provided with 1 l freshly collected cow manure for 24 h (exposed flies), while the other was maintained as before (unexposed flies) (**A**). Strawberry plants with bagged unopened flowers were placed into one of three cages, each containing four plants. The three cages were designated ‘exposed’, ‘unexposed’ and ‘control’. Five flies at a time were added to the appropriate exposed and unexposed cages, and the control cage remained free of flies for the duration of the experiment. As flowers opened, one at a time were unbagged and observed until they had had two minutes cumulative contact with flies, at which point the flowers were either collected and stored at − 20 °C or re-bagged to allow to develop to fruits. Once all flowers had been visited, flies were removed from the cages and euthanised by freezing at − 20 °C. Fruits were allowed to ripen in the bags before being collected and stored at − 20 °C. **B** For each of the exposed and unexposed groups of flies, 11 were taken for DNA extraction and sequencing, For the exposed, unexposed and control groups of flowers and developed fruits, between nine and 12 individuals were taken for DNA extraction and sequencing. Additionally, four cow manure samples were also sequenced (**C**)

Approximately 500 pupae of blow fly, *Calliphora stygia* (Fabricius 1782) (Calliphoridae), were obtained from a commercial supplier (Sheldon’s Baits, Parawa, South Australia) (Fig. 1). The fly pupae were roughly divided between two BugDorm™ 2F1M Cages (W60 x D60 x H60 cm) that were maintained in the same glasshouse as the strawberry plants. Fly pupae in both treatments were placed in a plastic takeaway container and covered in 5 cm of damp sand to facilitate eclosion from the puparium. Following eclosion, flies were provided with 60 ml of a 1:1 sucrose and distilled water solution. The sugar solution was provided in a clear plastic Petri dish with cotton wool balls pressed down into the sugar solution to facilitate landing and prevent the flies from becoming trapped in the liquid. The sugar solution was replaced daily. A laminated yellow cardboard disc was placed below the Petri dish to serve as a visual attractant and feeding stimulant to the flies. At all points in the experiment, efforts were made to minimise the likelihood of environmental contamination. All equipment used was pre-sterilised using bleach or ethanol where appropriate and handled with gloves that were changed when moving between cages. The adult flies were maintained in this way for 7 days prior to the start of the experiment.

At the start of the experiment, one cage of flies was randomly assigned as the contaminated treatment group, henceforth the “exposed flies” group. In this group, approximately 1 l of fresh cow manure was provided to the adult flies in a clean takeaway container. Cow manure was sourced from cows on the university farm at the Hawkesbury Campus. Flies rapidly accepted the cow manure, with all ~ 250 flies landing on it within seconds of being added to the BugDorm™ cage. The flies were left to forage on the cow manure for 24 h before the manure was removed. The flies that had not been provided with cow manure, henceforth the “unexposed flies” were maintained in the same way as before.

To allow the flies to access the flowers, flies were collected using sterile 50 ml tubes and transferred to the cages containing the strawberry plants. Five flies were released per cage (*n* = 5) and left to forage on the strawberry plants. The nylon mesh bags were removed from one open strawberry flower at a time. The flowers were observed and the durations of visits to the flowers were recorded. Visitation activities included walking across and around the flower, as well as frequent feeding bouts, in which the flies dabbed at the pistils and anthers with their labellum. Once each flower had received 2 cumulative minutes of visitation from the flies within the cages, the flowers were either collected or re-bagged and allowed to develop into fruits. In control flowers and fruit, nylon mesh bags enclosing flowers were not removed and hence did not receive visitation by either exposed or unexposed flies. All fly-flower visits were conducted within 48 h of the manure exposure. All flies were then removed from the cages. The fruits typically took around 21 days to fully develop and ripen. Flowers and fruits were placed into sterile tubes and frozen at − 20 °C prior to microbial DNA extraction and library preparation.

### DNA Extraction and Library Preparation

DNA from all samples was extracted using a Qiagen DNA Blood and Tissue kit with modified protocols. For flowers and flies, bacteria were dislodged from the surface of whole individuals by sonication in cold 1X phosphate-buffered saline (PBS) with 0.01% tween (1 ml and 500 µl respectively). Whole fruits were macerated in 1 ml of the cold PBS/tween solution. 100 µl of supernatants for flowers, fruits and flies, and 5 mg for cow manure, were then incubated with 20 µl proteinase K and 180 µl ATL lysis buffer for 1 h at 56 °C before continuing following manufacturer’s protocols.

DNA was quantified using fluorometry (Qubit) and transported on dry ice to the Ramaciotti Centre for Genomics, University of New South Wales, NSW, for library preparation using primers targeting the hypervariable V4 region of the 16 S ribosomal RNA gene (341 F and 805R [14], and sequencing on the Illumina Novaseq 6000 to generate 250 bp paired end reads. One no template control library was also prepared alongside the samples and sequenced.

### Data Analysis

Raw sequence reads (fastq format) were denoised using DADA2 (Callahan et al., 2016) and taxonomy was assigned to amplicon sequence variants (ASVs) by applying the classify-sklearn algorithm in QIIME 2 (v2021.2) using a Naïve Bayes classifier pre-trained on the Greengenes 13 − 8 database. The phylogenetic relationships between ASVs were determined in QIIME 2 through a multiple sequence alignment using MAFFT (Katoh & Standley, 2013) and phylogenetic reconstruction using fasttree (Price et al., 2009). QIIME data artifact (qza) files were then imported into Rstudio v4.3.2 (R Core Team, 2023) for subsequent analyses. These data were then converted to a *Phyloseq* object using *Phyloseq* v1.46.0 (McMurdie & Holmes, 2013) and the *Decontam* package v1.22.0 (Davis et al., 2018) was then used to identify and remove contaminant ASVs using the ‘prevalence’ method and following recommendations from (Díaz et al., 2021) to identify contaminants as all sequences more prevalent in controls than true samples. The dataset was then filtered further to remove mitochondria and chloroplast sequences and retain only bacterial ASVs using the subset_taxa command in the *Phyloseq* package. Rarefaction curves were generated for all samples, with the exclusion of the negative control, remaining after quality control and filtering using the ‘ggrare’ function in the *Ranacapa* package v0.1.0 (Kandlikar et al., 2018), followed by rarefaction according to plateauing of curves. The resulting rarefied counts table was then used for diversity analyses, with all other analyses being conducted on filtered, but not rarefied data.

Alpha diversity (Shannon’s index) was calculated using the *MicrobiotaProcess* package v1.14.0 (Xu et al., 2023) and plotted using *ggplot2* v3.5.2 (Wickham, 2011). Statistical significance between groups was calculated using Kruskal Wallace Rank Sum tests using the ‘kruskal.test’ function in the *stats* package v4.3.2 (R Core Team, 2023) with *post hoc* pairwise testing using Dunn’s tests with Bonferroni adjustment for pairwise testing (Dinno, 2017). Differences were considered statistically significant if *p* ≤ alpha/2. Beta diversity using the Bray-Curtis distance metric was calculated using the *Phyloseq* package with the ‘distance’ function, followed by ordination using the ‘ordinate’ function and plotting using ‘plot_ordination’. Ellipses were added to the plots using ‘stat_ellipse’ using the default 95% confidence levels assuming multivariate t-distribution. Overall differences in beta diversity between sample types, treatments and the nested effect of sample type:treatment were calculated using permutational multivariate analysis of variance (PERMANOVA) with the ‘adonis2’ function in the *vegan* package v2.6-4 (Oksanen J, 2022), with subsequent pairwise comparisons calculated using the ‘pairwise.adonis2’ function in the *pairwiseAdonis* package v0.4.1 (Arbizu, 2017). Differences between groups were considered statistically significant if *p* ≤ 0.05. To identify whether there were statistically significant differences between samples from the different treatment groups (exposed and unexposed for flies and exposed, unexposed and control for flowers and fruits), data were subset by sample type and distance metrics recalculated. For each sample type, ‘adonis2’ and ‘pairwise.adonis’ tests were again used to determine whether samples from the different treatments were statistically significant.

Relative abundance plots were created from the filtered *Phyloseq* object, with *ggplot2.* Determination of differentially abundant bacteria between the three treatments was carried out for the fly, flower and fruit sample types separately using the ‘ancombc2’ function in the *ANCOMBC* package v2.4.0 (Lin & Peddada, 2020, 2024). A heatmap showing the relative abundance of ASVs was generated using the ‘plot_heatmap’ function in the *Phyloseq* package. An upset plot was generated using the *UpsetR* package v1.4.0 [6] to visualise the number of ASVs shared by different sample groups and identify any ASVs which were present in the exposed group only, i.e., those that would have originated from the cow manure. Further, microbial source contributions to fruit-associated bacterial communities were then estimated using SourceTracker2 v2.0.1 [23]. As input, ASV tables were first filtered to remove rare taxa (present in fewer than two samples or with a total abundance < 10 reads across the dataset), then samples with < 100 sequencing reads were discarded. Source environments comprised manure, flies, and flowers, and fruit samples were designated as sinks. SourceTracker2 was run using the Gibbs sampler with default parameters, and sink samples were rarefied to 100 reads to prevent samples with higher sampling depths from dominating the contributions. Differences in proportional contribution within and among fruit treatments were assessed using Kruskal Wallace Rank Sum tests (‘kruskal.test’ function), with pairwise Wilcoxon signed-rank tests with Bonferroni correction used for subsequent pairwise testing (‘pairwise.wilcoxon.test’ function) using the *stats* package.

## Results

Exposure to manure affected both the microbial diversity of flies and the flowers they visited, but not the resulting fruits.

Altogether, 16 S rRNA gene amplicon sequencing was carried out for 91 samples comprising 11 exposed flies, 11 unexposed flies, 11 control flowers, 11 unexposed flowers, 12 exposed flowers, 10 control fruits, 9 unexposed fruits, 12 exposed fruits, four cow manure samples, and one negative control libraries (Fig. 1). After quality control, filtering, including removal of four ASVs flagged as potential contaminants, and inspection of rarefaction curves (Supplementary Fig. 1), four samples with fewer than 101 reads were removed. Given not all sample types’ rarefaction curves plateaued at this depth, we experimented with increasing rarefaction depths for diversity analyses. This showed no meaningful differences in results, but confidence in the within sample type results was decreased due to lower sample sizes, especially for fruit samples. Therefore, we opted to use a rarefaction depth of 102 reads for the diversity analyses, but used un-rarefied data for all other analyses. The final dataset comprised an average of 19,384 reads per sample, ranging from 102 to 117,089 (Supplementary table S1).

**Fig. 2:**
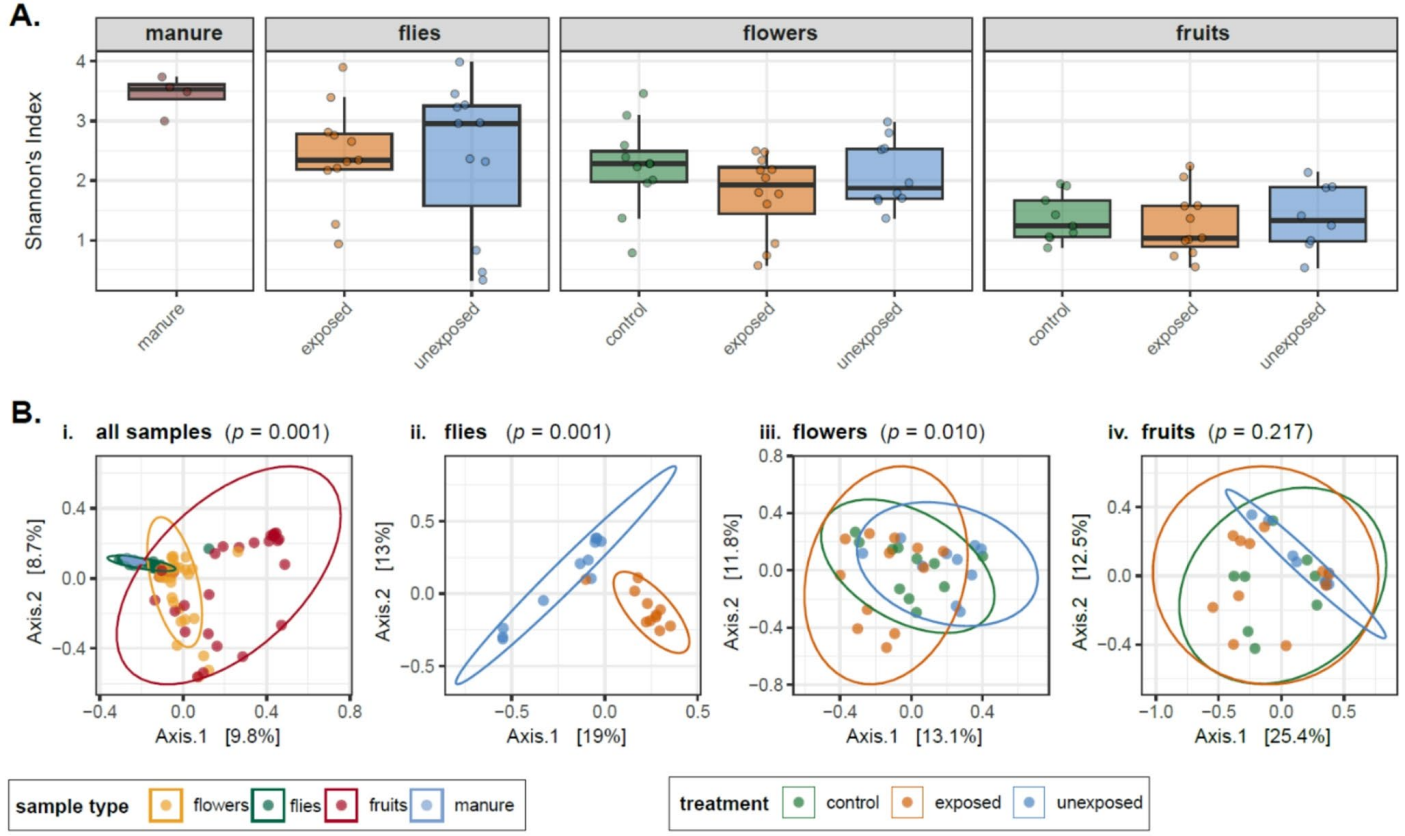
Diversity analyses, grouped by sample type and treatment. **A** Alpha diversity of samples from each treatment type, facetted by sample type. The fruits and flowers comprise samples belonging to the three sample types: exposed, unexposed and control (self-pollinated). The fly samples comprised individuals that had or had not been provided with cow manure (exposed and unexposed) and cow manure samples were also sequenced. **B** Beta diversity calculated using the Bray Curtis dissimilarity metric and visualised with PCoA plots. **i**) all samples analysed together, coloured by sample type, **ii-iv**) samples were then subset according to sample type and coloured according to treatment. *p* values show results of PERMANOVA analyses to determine differences between **i**) sample types and **ii-iv**) treatments analysing each sample type separately

Overall, alpha diversity (Shannon’s Index) was significantly different between sample types (Kruskal-Wallis, ꭕ^2^ = 29.47, *p* = < 0.001), with the fruit samples being less diverse than all other sample types (Dunn’s test, *p* = < 0.003 for all pairwise comparisons). After sub-setting by sample type, there were no statistically significant differences when comparing diversity between treatments (control, unexposed, exposed) for flies, flowers or fruits (Fig. 2A).

In terms of beta diversity, however, overall there was significant clustering by sample type, treatment and an interaction effect of sample type and treatment (adonis *p* ≤ 0.001 for all variables, Fig. 2Bi, Supplementary table S2). Analysing sample types separately, fly samples grouped together according to whether they had been exposed to cow manure or not (adonis *p* ≤ 0.001, Fig. 2Bii, Supplementary table S2). With the flowers, the unexposed and exposed treatments were significantly different (adonis *p* ≤ 0.003), but neither group was different to the control (Fig. 2Biii, Supplementary table S2). At the fruit stage, there was no longer any difference between treatments (Fig. 2Biv, Supplementary table S2).

**Fig. 3:**
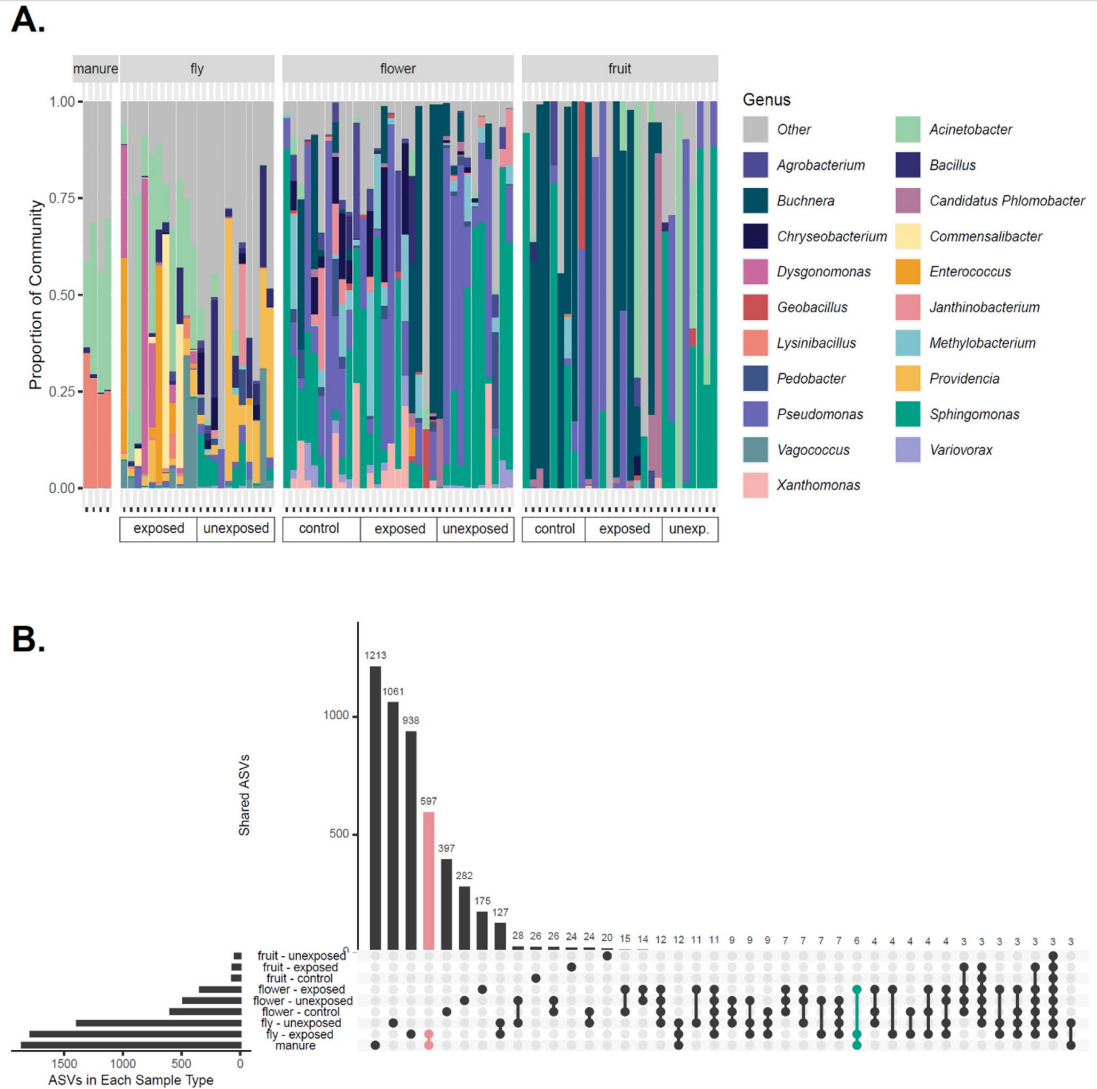
Taxonomic composition of each sample type. **A** Relative abundance of genera in each sample, showing the top 20 genera across the dataset with all other taxa grouped as ‘Other’, faceted by sample type (manure, fly, flower, fruit) and ordered by treatment (control, exposed, unexposed). **B** Upset plot showing the number of amplicon sequence variants (ASVs) in each of the nine sample type categories as horizontal bars, for example the total number of ASVs detected in the combined manure samples was 1859. Balls and sticks represent intersections where ASVs were detected in multiple sample categories, with vertical bars showing the number of ASVs in each intersection, for example there were 597 ASVs that were present only in the manure and ‘fly-exposed’ sample groups (highlighted in pink) and 6 ASVs that were present in the manure, ‘fly–exposed’ and ‘flower–exposed’ groups (highlighted in green). The sum of the vertical bars equals 5115 ASVs, the total number present in the dataset

Composition of fly and, to a lesser extent flower, microbiome is influenced by fly exposure to cow manure, but manure-derived ASVs do not persist to the fruit stage. Overall, the microbiome composition at genus level varied across sample types, with the manure samples being dominated by *Lysinibacillus* and *Acinetobacter*, flies harbouring more *Providencia*, and flowers and fruits containing more *Sphingomonas* and *Pseudomonas* (Fig. 3A). While all sample types, except for manure, showed considerable individual variation, within the fly samples there was a notable difference between the exposed and unexposed individuals, with the exposed flies containing significantly more *Acinetobacter*; a dominant genus in the manure samples (Ancom-bc2, *p* = 0.0001). In particular, there were two *Acinetobacter* ASVs that were present in every manure sample and every exposed fly sample, but these were present in only 0/11 and 3/11 unexposed flies (Supplementary figure S2). The flower and fruit microbiomes, however, were more similar across treatments and showed no statistically significant differences in the abundance of genera between any of the three treatments. Given the nature of 16 S rRNA data, it was not possible to identify taxa beyond the genus level, so unfortunately, we were unable to assess whether pathogen sequences were present in the dataset.

Across the whole dataset, a total of 5115 ASVs were detected (Fig. 3B). The manure samples contained a total of 1859 ASVs, with approximately one third of these (1213) not being found in any other sample type. There were 597 ASVs which were exclusively found in the manure and exposed fly samples. However, only 6 ASVs were shared between manure, exposed fly and exposed flower samples: three ASVs identified at the genus level, *Acinetobacter*, *Clostridium* and *Pseudobutyrivibrio*, one in the family Pseudomonadaceae, one in the order Bacillales, and one ASV in the class Mollicutes. There were no ASVs which were found in both the manure and exposed fruit samples.

Source tracker analyses indicated that across fruit samples, inferred contributions differed significantly among source environments (Kruskal-Wallis, ꭕ^2^ = 61.116, *p* = < 0.001), with flowers contributing significantly more than flies or unknown sources, and manure contributing minimally (Wilcoxon signed-rank tests, *p* = < 0.001 for all pairwise comparisons with the exception of the fly-unknown comparison which was not statistically significant (*p* = 0.345)). In contrast, the proportional contribution of each source did not differ significantly among fruit treatments (control, exposed, unexposed, Kruskal Wallis test *p* > 0.05 for all sources (Supplementary figure S3), further supporting a lack of treatment effect on fruit microbiome composition.

## Discussion

Here, we aimed to respond to grower and consumer concerns over the risks to food safety of using blow flies as alternative pollinators. Through conducting a glasshouse pollination experiment, we found that while blow fly visitation affected the microbial composition of strawberry flowers, differences did not persist to the fruit stage. In particular, when flies had been allowed contact with cow manure prior to pollination, no amplicon sequence variants (ASVs) could be tracked through from manure, to fly and flower, all the way to developed fruit.

Previous work has shown that as strawberry fruits develop, microbiome members remain on developing fruit after pollination [34]. Accordingly, here we saw an overlap in microbiome composition between flowers and fruits, with *Sphingomonas* and *Pseudomonas* common dominant members, and *Methylobacterium* and *Variovorax* frequently seen as minor components, as observed previously [34, 44, 51]. In addition, source tracking analysis also showed that flower-associated bacteria were the dominant source contributing to the fruit microbiome.

Much of the blow fly microbiome is acquired from their environment, including from foraging on dead and decaying organic matter [18]. These bacteria can subsequently be transported and deposited on other surfaces, including flowers. The microbiome of blow fly species differs between external and internal body parts [8, 20]. Here we focussed on the external microbiome, with the hypothesis that these would be the bacteria most likely to be transferred to flowers. Flies from both exposed and unexposed groups contained previously observed genera including *Acinetobacter*, *Providencia*, and *Vagococcus* [8, 20]. Striking differences were seen between the exposed and unexposed flies, however, with the exposed flies harbouring considerably more *Acinetobacter*, which was also a dominant member of the manure microbiome. This genus has been shown previously to be shared between flies and their food substrate, suggesting it may be particularly amenable to substrate to fly transfer and persistence [18]. Furthermore, pathogenic *Acinetobacter baumanii* strains have previously been isolated from manure samples [40] suggesting the transferred bacteria could potentially comprise pathogenic species. Although it is not possible to confidently assign species level information to 16 S rRNA data, a blastn search of *Acinetobacter* ASVs did not produce any hits to *A. baumanii* and this genus also comprises many non-pathogenic species. Overall, 33.4% (597/1788) of the ASVs in the exposed flies were also found in the manure samples, in contrast to the unexposed flies which only shared 0.2% (3/1389) of their ASVs with manure.

Given the apparent degree of bacterial transfer from manure to fly, it was then perhaps surprising to see far less transfer between flies and the flowers they visited. This could be due to the flies spending comparatively less time in contact with the flowers than the manure, the flowers maintaining lower bacterial abundance than the manure, or due to the bacteria being unable to persist on the flower surfaces. At the fruit stage there were no differences between control, unexposed and exposed flies, showing that at this point environmental filtering was the dominant factor in determining microbiome composition.

Our experimental setup only assessed fly deposition on the flower itself. In a field scenario, it is possible that flies may also land on the developing fruits, for instance, if small defects on the fruit made them more attractive to flies, and direct deposition for saliva or defecation would then also be possible. However, given that fruits provide few or no resources (nectar or pollen) to pollinators, blow flies are unlikely to make visits to them. Furthermore, blow fly visual systems are not strongly sensitive to red light wavelengths, like that of a ripe strawberry fruit [28]. Thus, the direct risk of flies visiting and contaminating fruit may be minimal.

While the results presented suggest a limited risk of bacterial transfer to fruits following blow fly pollination, we acknowledge the limitations of our study. We chose to use cow manure as blow flies commonly feed on this substrate, but we did not conduct any prior testing to ascertain whether potentially pathogenic bacteria were present in the manure. As transfer of pathogens is a primary cause of concern for growers, another approach here would be to use a specific pathogenic strain of bacteria such as *E. coli* or *Salmonella*. Whilst this alternative methodology may better reflect a real-world scenario, it may only be relevant to a very limited number of all the potentially harmful bacterial strains that could be present within the agri-food system. We also acknowledge that here we focussed only on bacteria, while other components of the microbiome, including yeasts, are also commonly associated with fruits and flowers, and pathogenic yeasts have previously been found on fruit surfaces [5]. Furthermore, as we focussed on the external fly microbiome, we may have missed any bacteria present in the fly gut or saliva which could also have been transferred to flowers during pollination. Given there were no ASVs shared exclusively between manure, exposed flowers and exposed fruits, it seems unlikely that specific microbes were picked up from the manure and deposited on the flower via saliva or faeces in this experiment. However, future studies examining gut and saliva contents could provide definitive proof as to whether potentially harmful microbes may be deposited in this way.

Strawberries are commonly transported, stored and washed prior to consumption, which may result in further microbiome changes. Whilst we did not test for any post-harvest changes to the strawberry microbiome, previous studies have found changes in composition and load over time [34, 51]. It is possible therefore, that if there are bacteria currently present but below the limits of detection, these have the potential to increase in abundance following a period of storage. However, storage has been found to have a minimal effect on pathogen persistence in strawberries [11, 24]. Furthermore, while washing fruits can reduce or remove pathogens from the fruit surface [46], this may not eliminate the bacteria as many of the bacteria may also reside within the pulp [34].

## Conclusions

Blow flies represent attractive alternative pollinators to honey bees. However, due to their associations with decaying matter including carrion and faeces, there is a perceived increased risk of contamination of fruits with pathogenic bacteria. Here we showed that while blow flies did pick up bacteria from manure, only a small subset of bacteria was transferred to the flowers during pollination. Further, there was no difference in fruit microbiome composition between any of our fly pollination treatments. As such, the food safety risk of using blow flies for strawberry pollination is likely to be minimal.

## Supplementary Information

Below is the link to the electronic supplementary material.

Supplementary Material 1

Supplementary Material 2

## Data Availability

All raw sequence reads (.fastq format) are publicly available at Sequence Read Archive under project id PRJNA1297051. Accessions for individual samples are provided in Supplementary Table 1. Scripts for all analyses and figure generation, as well as underlying data (phyloseq objects) are available at [https://github.com/laura-brettell/fly_16S](https:/github.com/laura-brettell/fly_16S) under Zenodo ID: [DOI: 10.5281/zenodo.17582175](https:/doi.org/10.5281/zenodo.17582175) .
