# Supplementary material for: Filth Flies, Flowers and Food: Pollination by Flies (Calliphoridae) Does Not Affect the Strawberry Microbiome
Source: Microb Ecol. 2026 Mar 25;89:93. doi: 10.1007/s00248-026-02741-2 (PMC13053414; doi:10.1007/s00248-026-02741-2)
Supplement: Supplementary file 1 [file 248_2026_2741_MOESM2_ESM.docx]

Filth flies, flowers and food: pollination by flies (Calliphoridae) does not affect the strawberry microbiome

Jonathan T. D. Finch^1,2,#^, Markus Riegler^1^, James M. Cook^1^, Laura E. Brettell^1,3,#^

Supplementary Information

**Figure S1:** Rarefaction curves showing species richness per sample, facetted by sample type, up to a depth of 101 reads per sample, as used for rarefying for diversity analyses.

**Figure S2:** Heat map showing the relative abundance of ASVs in each sample, including all ASVs present at ≥ 5% relative abundance in at least one sample. Each row corresponds to a single ASV and is labelled on the y axis according to genus if known or, if unknown, the lowest taxonomic ranking known. Each column corresponds to a single sample, faceted by sample type and grouped by treatment.

**Figure S3:** Boxplots showing proportional contributions of flower, fly, and manure-associated bacterial communities, as well as an unknown source, to fruit samples belonging to control, exposed, and unexposed treatments estimated using *SourceTracker2*. Boxes represent interquartile ranges with medians indicated by horizontal lines. Differences in source contributions among fruit treatments were assessed using Kruskal–Wallis tests and no significant differences were detected (*p* > 0.05).

**Table S1:** Sample metadata including read numbers per sample after filtering and per sample Sequence Read Archive accessions. (separate file)

**Table S2:** Beta diversity results, both overall per sample type and comparing treatments within sample types. (separate file)
